# Isolation, identification, and characterization of plant growth-promoting bacteria from Pisha sandstone for application as a microbial inoculant in soil rehabilitation

**DOI:** 10.3389/fmicb.2025.1664490

**Published:** 2025-10-07

**Authors:** Xiuwen Fang, Haiying Gao, Zhishui Liang

**Affiliations:** ^1^School of Civil Engineering, Southeast University, Nanjing, China; ^2^Key Laboratory of Concrete and Prestressed Concrete Structures of the Ministry of Education, Southeast University, Nanjing, China; ^3^National and Local Unified Engineering Research Center for Basalt Fiber Production and Application Technology, Southeast University, Nanjing, China

**Keywords:** Pisha sandstone, plant growth-promoting bacteria, soil fertility, ecological restoration, microbial inoculant

## Abstract

Pisha sandstone is a loosely cemented sedimentary rock widely distributed in the Yellow River Basin and the Ordos Plateau. Due to its high montmorillonite content (20–30%) and low quartz content (approximately 50%), it rapidly disintegrates upon water exposure, resulting in severe soil erosion and contributing to the major coarse sediment in the mid-upper reaches of the Yellow River. Additionally, its low mechanical strength and poor fertility limit the effectiveness of traditional conservation approaches. In this study, an eco-friendly plant-microbe strategy for restoring degraded Pisha sandstone areas was proposed. Plant growth-promoting bacteria were isolated from the rhizosphere of *Medicago sativa* and reclaimed coal-mining lands in different Pisha sandstone areas. Of 97 isolates, five bacterial strains (F11, F6, G1, G3, P9) demonstrated significant activity in phosphate solubilization, nitrogen fixation, indole-3-acetic acid (IAA) biosynthesis, and siderophore production. They were identified as Klebsiella, Acinetobacter, Herbaspirillum, Pseudomonas, and Serratia. Pot experiments using *Medicago sativa* and *Astragalus adsurgens* in Pisha sandstone substrates revealed that bacterial inoculation significantly improved soil organic matter, available nitrogen, phosphorus, and potassium, and enhanced plant height and biomass production. The microbial agent formulated with strains F11 and G1 (MI3) exhibited the optimal performance. These results highlight the potential of plant-microbe interactions for ecological restoration of fragile sandstone ecosystems and offer a sustainable strategy for controlling soil degradation in the Yellow River Basin.

## 1 Introduction

Pisha sandstone is a typical loosely consolidated rock formation widely distributed in the transitional zone where Shanxi Province, Shaanxi Province, and the Inner Mongolia Autonomous Region converge, located within the Yellow River basin and the Ordos Plateau region of northern China. Its mineral composition is primarily composed of quartz, feldspar, calcite, montmorillonite, kaolinite, illite, and biotite. Among these, the mass fraction of quartz is just about 50%, which is significantly lower than that of typical sandstone (Shi et al., 2004; Liang et al., 2019; Zhao et al., 2023). Montmorillonite is the second most abundant mineral in Pisha sandstone after quartz, accounting for approximately 20 to 30% of its total mineral composition. Montmorillonite can expand to more than 150% of its dry volume upon water absorption, leading to internal structural disintegration and making the rock highly susceptible to collapse in water infiltration environments (Wang et al., 2021). Due to this characteristic, severe erosion frequently occurs following rainfall events in this region (Liang et al., 2020). Although this area accounts for only approximately 2% of the total area of the Yellow River Basin, it is a major source of coarse sediments in the upper reaches of the Yellow River, accounting for 25% (Zhang et al., 2009). As a result, it has been referred to as an “environmental cancer” (Ni et al., 2008).

Intense water erosion in the Pisha sandstone region has led to significant degradation of the ecological substrate, manifesting as both nutrient deficiency and physical soil deterioration. This combination of stresses poses a significant constraint on the establishment and sustained growth of most plant communities (Liang et al., 2024). This persistent ecological stress ultimately hinders vegetation development, leading to low vegetation coverage and extensive surface exposure (Wang et al., 2020). To mitigate soil erosion, three main types of measures have been adopted: engineering, biological, and material consolidation methods. Engineering measures mainly include gully head protection, slope stabilization, and gully engineering. Due to its low degree of diagenesis, weak structural strength, and high susceptibility to disintegration when exposed to water, Pisha sandstone exhibits poor resistance to hydraulic scouring. As a result, it is not suitable for direct use as a construction material for sediment retention dams, which significantly hinders the advancement of such engineering projects in the region (Yao et al., 2019). Biological measures primarily involve the use of sea buckthorn to form “soft dams” through vegetation. However, the extremely thin soil layers and low water retention capacity of Pisha sandstone slopes make vegetation restoration difficult and result in limited short-term effectiveness (Xiao et al., 2014). Currently, the most effective material-based method involves the use of modified hydraulic polyurethane (W-OH) to consolidate Pisha sandstone, which can enhance vegetation growth and coverage while improving soil water and nutrient retention capacities (Ma et al., 2020; Liang et al., 2024). Nevertheless, this approach is generally limited to small-scale applications and faces several implementation challenges.

In recent years, increasing attention has been paid to the role of plant-microbe interactions in soil improvement and ecological restoration. Plant roots secrete organic acids, sugars, and other carbon sources that stimulate rhizosphere microbial activity. In turn, microbes contribute to nutrient cycling by processes such as nitrogen mineralization, phosphatase hydrolysis, and manganese redox reactions (Khoso et al., 2024). Moreover, plant growth-promoting bacteria (PGPB) enhance plant tolerance to abiotic stresses through multiple mechanisms, including the production of ACC deaminase to reduce stress ethylene levels, the release of phytohormones such as indole-3-acetic acid (IAA) and cytokinins to regulate plant responses, and the synthesis of protective metabolites such as proline and trehalose, which mitigate drought and salt stress (Gamalero and Glick, 2022). Beyond stress tolerance, phosphate-solubilizing bacteria transform insoluble phosphorus into plant-available forms through acidification and enzymatic hydrolysis, thereby significantly improving the fertility of reclaimed soils (Ahmad et al., 2022). Beyond stress tolerance, phosphate-solubilizing bacteria transform insoluble phosphorus into plant-available forms through acidification and enzymatic hydrolysis, thereby significantly improving the fertility of reclaimed soils (Kong and Glick, 2017; Li et al., 2025). Furthermore, photosynthetically derived carbon compounds from plants are continuously deposited in the rhizosphere, supplying energy to microbial communities and establishing a positive feedback loop between plants and microbes (Farrell et al., 2020; Zhang et al., 2021; Li et al., 2022).

Previous studies provide important references for this research. For example, Li L. et al. (2020) found that in reclaimed soils affected by coal mining subsidence and human disturbances, traditional chemical phosphorus fertilizers were easily immobilized by the soil, resulting in low phosphorus availability. In contrast, phosphate-solubilizing bacteria significantly improved the availability of various forms of inorganic phosphorus through acidification, enzymatic hydrolysis, and protein pathways, thereby enhancing the nutrient supply capacity of the soil. Wu et al. (2024) reported that microbial inoculants can enhance soil quality under saline-alkali stress by increasing rhizosphere microbial diversity and network complexity, enzyme activity, and nutrient release efficiency. Li et al. (2025) isolated and combined functional microbial communities from degraded mining soils and demonstrated their key role in enhancing the resilience of plant–soil systems to environmental stress. These studies collectively indicate the feasibility and broad application potential of ecological restoration strategies based on plant-microbe synergistic interactions.

In addition, both *Medicago sativa* and *Astragalus laxmannii* are naturally distributed in the Pisha sandstone region. Among them, *Medicago sativa* has been widely used in both pot and field experiments, and is considered a representative species for vegetation restoration owing to its strong adaptability and soil-improving effects in this fragile environment (Jia et al., 2019; Liang et al., 2024). By contrast, *Astragalus laxmannii* is also a native legume in the Pisha sandstone region, but it has mainly been recorded in vegetation surveys, with few systematic experimental studies conducted to date.

Based on this context, the present study employed a controlled pot experiment to evaluate the short-term feasibility of microbial inoculants in promoting the early growth of *Medicago sativa* and *Astragalus laxmannii* under the nutrient-deficient conditions of Pisha sandstone. Although the mechanisms of plant–microbe synergistic soil improvement have been validated in reclaimed and saline-alkali lands, their applicability to Pisha sandstone remains largely unexplored. To address this gap, we aimed to isolate plant growth-promoting bacteria (PGPB) from the rhizosphere soil of *Medicago sativa* in Pisha sandstone regions, coal mine reclamation areas, and exposed Pisha sandstone soils. Combined with stress-tolerant plants such as *Astragalus laxmannii* and *Medicago sativa*, plant–microbe consortia were constructed to explore their potential in improving Pisha sandstone structure and enhancing soil fertility. This work seeks to propose a cost-effective, eco-friendly, and sustainable strategy for Pisha sandstone remediation, providing theoretical support and practical guidance for desertification control and ecological restoration in the Yellow River Basin.

## 2 Materials and methods

### 2.1 Soil and plant materials

Soil samples for microbial isolation were collected from three distinct sources in Zhungeer Banner, Ordos City, Inner Mongolia, China: (1) surface soil from the exposed Pisha sandstone area, (2) rhizosphere soil of wild *Medicago sativa* growing naturally in the same region, and (3) reclaimed soil from the Juxinlong Coal Mine restoration site (110°4′2″E, 39°54′16″N). All samples were placed in sterile zip-lock bags and transported to the laboratory under cooled conditions.

The Pisha sandstone soil used for the pot experiment was also collected from the Pisha sandstone area in Zhungeer Banner and air-dried in a shaded, well-ventilated indoor environment. After drying, the soil was sieved through a 5 mm mesh and stored for further use.

**Figure 1:**
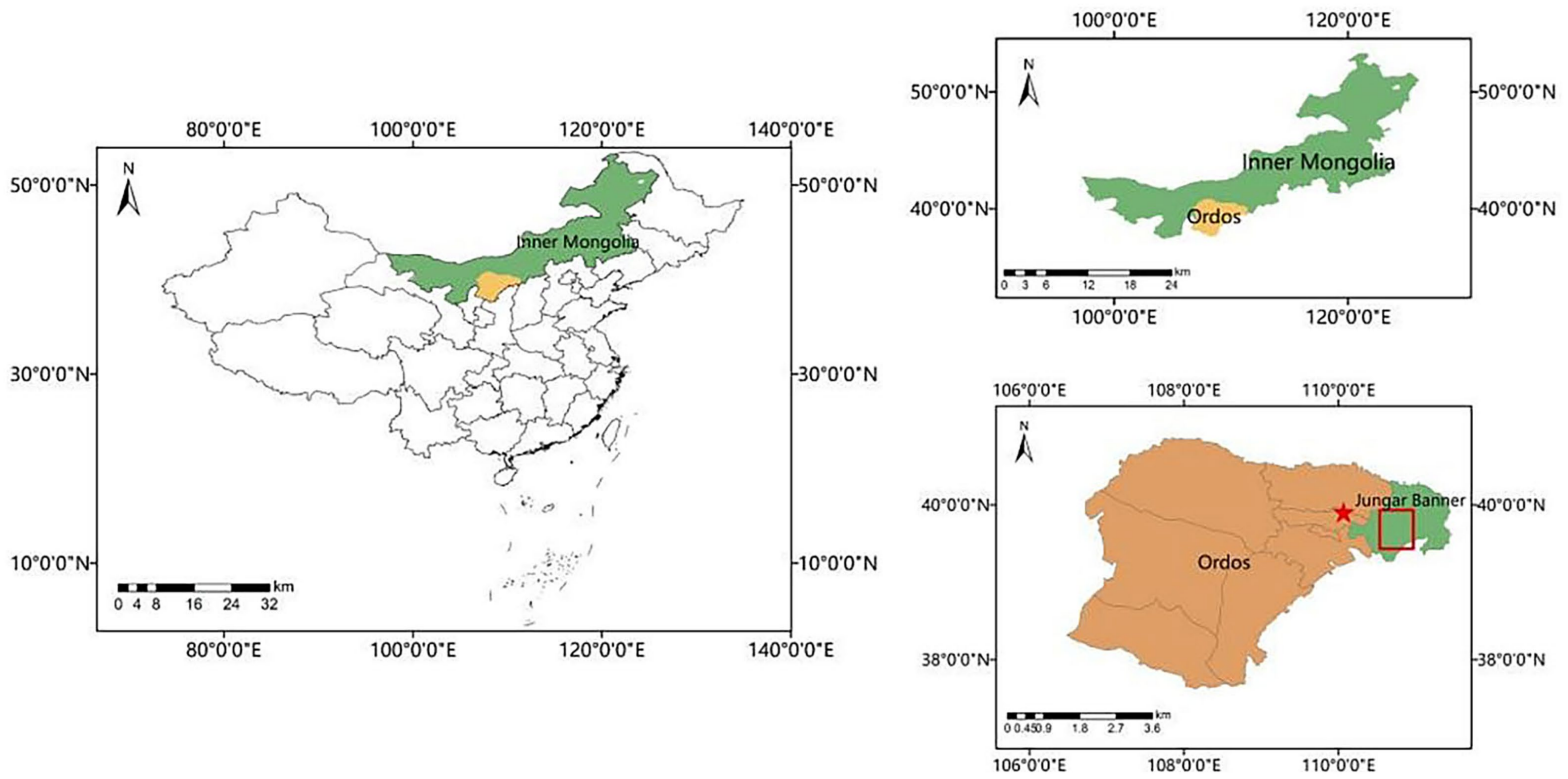
Sampling locations.

Specifically, the surface soil, rhizosphere soil from wild *Medicago sativa*, and the potting soil for the greenhouse experiment were all sourced from the Erlahugou watershed region in Zhungeer Banner, Ordos City (110°32′-111°06′E, 39°26′-39°56′N), as shown in Figure 1. In contrast, the reclaimed soil was collected from a separate restoration site.

The seeds of *Medicago sativa* and *Astragalus laxmannii* were purchased from Ordos City, Inner Mongolia, China, and used as the plant materials in this study.

### 2.2 Experimental design

Isolated bacterial strains were quantitatively evaluated for their capacities in solubilizing organic and inorganic phosphate, nitrogen fixation, siderophore production, and indole-3-acetic acid (IAA) synthesis. Based on a comprehensive assessment of these functional traits, the more promising strains were selected and formulated into bacterial suspensions.

Seeds of *Medicago sativa* and *Astragalus laxmannii* were surface-sterilized by immersion in 5% sodium hypochlorite solution for 10 min, then rinsed three times with sterile distilled water. The sterilized seeds were evenly spread on moist sterile gauze and incubated at 30 °C for 24 h. Once germinated (i.e., radicles emerged), the seeds were sown in pots containing 400 g of Pisha sandstone soil, with 15 seeds per pot. Each treatment included application of the bacterial suspension at 5% (v/w) soil ratio. Control check (CK) received an equal volume of sterile distilled water instead of bacterial suspension. Each treatment was performed in triplicate.

The first round of pot experiments was conducted in a greenhouse starting on 16 February 2025, and above-ground plant parts and rhizosphere soil were harvested after 30 days on 18 March 2025. Soil samples were analyzed for organic matter, available nitrogen, phosphorus, and potassium, while plant height and fresh weight were also recorded. After approximately 7 days of data measurement and processing, the most effective bacterial strains were selected to produce microbial inoculants.

The second round of pot experiments with the prepared microbial inoculants was then conducted under the same greenhouse conditions, starting on 25 March 2025. Microbial inoculants were mixed with Pisha sandstone soil at a 5% (w/w) ratio (400 g per pot), with each treatment repeated three times. The control group (CK1) received only the inoculant carrier without microbes at the same ratio. Seeds were sterilized, germinated, and sown into the pots as described above. After 30 days, on 24 April 2025, the aerial parts of the plants were harvested, and rhizosphere soil samples were collected for subsequent analysis.

### 2.3 Culture media

LB medium was used for routine bacterial cultivation. Potassium-solubilizing medium, inorganic phosphate medium, organic phosphate medium, IAA medium, CAS medium, and nitrogen-fixing medium were used to assess the potassium-solubilizing ability, inorganic phosphate solubilization, organic phosphate solubilization, IAA production, siderophore production, and nitrogen-fixing capacity of rhizobacteria, respectively.

### 2.4 Isolation and screening of soil bacteria

Approximately 1 g of rhizosphere soil from wild *Medicago sativa*, surface soil from the Pisha sandstone region, and soil from the reclaimed Juxinlong Coal Mine area were weighed and added to Erlenmeyer flasks containing 99 mL of sterile water. The mixtures were shaken at 160 rpm for 30 min. Then, 1 mL of the suspension was transferred into a test tube containing 9 mL of sterile water and thoroughly mixed. Serial dilutions were prepared to obtain dilutions ranging from 10^−3^ to 10^−7^. Aliquots (100 μL) of each dilution were spread onto LB agar plates and incubated at 28 °C for 72–96 h until colonies appeared. A total of 53 bacterial isolates were obtained and streaked on LB agar plates to obtain pure cultures. These pure cultures were then transferred to solid organic phosphate, inorganic phosphate, and nitrogen-fixing media. Strains that were able to grow after three successive transfers on nitrogen-fixing medium and produced large clear halos on phosphate media were selected. A total of 8 such strains were obtained and preserved in 30% glycerol at −20 °C for further use.

### 2.5 Quantitative determination of nitrogenase, phosphate solubilization, IAA, and siderophore production

A total of 8 such strains were obtained and preserved in 30% glycerol at −20 °C for further use. The nitrogen-fixing ability of the isolated strains was assessed using a microbial nitrogenase ELISA detection kit, with nitrogenase activity serving as an indicator of nitrogen fixation capacity.

Phosphate solubilization ability was assessed using the molybdenum blue method (Murphy and Riley, 1986; Ait-Ouakrim et al., 2023). Bacterial suspensions (OD600 = 1.0) were inoculated into NBRIP inorganic/organic phosphate liquid media and incubated at 28 °C with shaking for 3 days. After centrifugation, 0.5 mL of supernatant was mixed with 10% trichloroacetic acid and a molybdate-ascorbic acid-sulfuric acid reagent. The mixture was incubated in the dark for color development, and absorbance was measured at 820 nm. Soluble phosphate concentration was calculated based on a KH₂PO₄ standard curve. This method quantifies phosphate solubilization by detecting the blue-colored phosphomolybdenum complex formed after reduction with ascorbic acid (Ducousso-Détrez et al., 2024).

IAA production was determined using the Salkowski method (Glickmann and Dessaux, 1995; Kapadia et al., 2022). Bacterial strains were cultured in LB broth supplemented with 200 mg/L tryptophan at 30 °C and 180 rpm for 3 days. After centrifugation, equal volumes of the supernatant and Salkowski reagent were mixed and incubated in the dark for 30 min. Absorbance was measured at 530 nm.

Siderophore production was evaluated by inoculating equal volumes of bacterial suspension into Modified Katznelson and Bose (MKB) broth, a medium commonly used to assess the phosphate-solubilizing potential of microorganisms. The cultures were incubated at 28 °C with shaking at 150 rpm for 48 h. After incubation, the cultures were centrifuged at 10,000 rpm for 10 min to obtain the supernatant. A 3 mL aliquot of the supernatant was thoroughly mixed with 3 mL of Chrome Azurol S (CAS) assay solution and left to stand in the dark for 1 h. Absorbance was then measured at 630 nm using a Model 721 spectrophotometer, and the result was recorded as As. Double-distilled water was used to zero the spectrophotometer. For the reference measurement (Ar), 3 mL of CAS assay solution was mixed with 3 mL of the supernatant obtained from uninoculated MKB broth and analyzed under identical conditions (Li et al., 2019). Siderophore production was calculated using the formula: Siderophore unit = (1-As/Ar) × 100% (Mustapha et al., 2024).

### 2.6 Molecular identification and phylogenetic analysis

Genomic DNA of the bacterial strains was extracted using the phenol-chloroform method. DNA purity was assessed using a NanoDrop ND-200 spectrophotometer, with acceptable samples exhibiting an A260/A280 ratio of approximately 1.8. Qualified DNA samples were stored at −20 °C. The 16S rRNA gene was amplified using the universal primers 27F (5′-AGAGTTTGATCCTGGCTCAG-3′) and 1492R (5′-GGTTACCTTGTTACGACTT-3′). The PCR protocol was as follows: initial denaturation at 94 °C for 5 min; 30 cycles of denaturation at 94 °C for 30 s, annealing at 55 °C for 30 s, and extension at 72 °C for 1.5 min; followed by a final extension at 72 °C for 7 min. The PCR products were sequenced bidirectionally, and sequence similarity searches were conducted using NCBI BLAST to determine the taxonomic affiliation of the strains. Multiple sequence alignments were performed using CLUSTAL-W in MEGA 11.0, and a phylogenetic tree was constructed by the neighbor-joining (NJ) method with 1000 bootstrap replicates. Phylogenetic relationships were inferred based on evolutionary distances and bootstrap support values of clade nodes (Injamum-Ul-Hoque et al., 2024).

### 2.7 Preparation of strain carrier, bacterial suspensions of strains, and microbial inoculants

The strain carrier was composed of sheep manure and straw-derived biochar mixed at a mass ratio of 2:1. For the preparation of bacterial suspensions, each isolate was cultured individually in LB liquid medium for 24–48 h, centrifuged at 10,000 rpm for 10 min, and resuspended in sterile distilled water to an OD_600_ of 1.0. For single-strain treatments, these suspensions were directly applied to the soil.

For the composite microbial inoculant, equal volumes of the suspensions of selected strains were mixed, after confirming compatibility through co-cultivation assays on LB agar where no antagonistic effects were observed. The resulting mixed suspension was combined with the carrier at a volume-to-weight ratio of 1:3, and the mixture was dried in an incubator at 30 °C to obtain the final inoculant product. The CK1 control carrier was prepared by adding an equal volume of sterile LB medium (without bacteria) to the carrier and drying under the same conditions.

### 2.8 Analysis of soil physicochemical properties and enzyme activities

Soil organic matter was determined by the dilution heat method; available phosphorus was extracted by sodium bicarbonate and quantified with a spectrophotometer; available potassium was extracted by ammonium acetate and quantified by a flame photometer; available nitrogen was determined by the alkaline diffusion method.

Urease activity was determined by quantifying NH_4_^+^ released from urea hydrolysis. Soil samples (1 g) were incubated with 50 mM urea in borate buffer (pH 10.0) at 37 °C for 2 h. After terminating the reaction with 1 M KCl and filtration, NH_4_^+^ concentration was measured colorimetrically at 690 nm using a sodium dichloroisocyanurate-salicylate/NaOH chromogenic system, with activity expressed as μg NH_4_^+^-N per gram dry soil per hour (Wan et al., 2025). Sucrase activity was evaluated based on glucose production via the 3,5-dinitrosalicylic acid (DNS) method. Air-dried soil (5 g) was mixed with 15 mL of 8% (w/v) glucose solution, 5 mL phosphate buffer (0.2 M, pH 5.5), and toluene (as microbial inhibitor), followed by incubation at 37 °C for 24 h. The filtrate (1 mL) was mixed with 3 mL DNS reagent and heated in a boiling water bath for 5 min. Absorbance of the cooled mixture was measured at 550 nm, and activity was calculated as mg glucose produced per gram of soil per 24 h (Zhang et al., 2024). Alkaline phosphatase activity was determined using a soil alkaline phosphatase (S-ALP/AKP) detection kit according to the manufacturer’s protocol.

### 2.9 Statistical analyses

The experimental results were processed and visualized using Microsoft Excel 2021 and Origin 2023. Statistical significance of the observed differences was evaluated through Duncan’s multiple range test, performed with SPSS Statistics version 25.0.

## 3 Results

### 3.1 Isolation and screening of plant growth-promoting bacteria from soil samples

A total of 97 bacterial isolates were obtained using the dilution plating method. Among them, 53 strains exhibiting vigorous growth and prominent colony morphology were selected for quantitative assessment of nitrogen fixation and phosphate solubilization abilities. These 53 isolates were subsequently transferred onto Ashby nitrogen-free solid medium, NBRIP inorganic phosphate solid medium, and NBRIP organic phosphate solid medium.

Strains that maintained stable growth after three successive transfers on Ashby medium and formed prominent clear halos on both NBRIP phosphate media were retained. Eight strains-P12, P9, F6, F11, F7, G1, G3, and G16-were selected for further study. Specifically, strains P12 and P9 were isolated from surface soil of the Pisha sandstone area; F6, F11, and F7 were obtained from reclaimed soils of the Juxinlong Coal Mine in Ordos; and G1, G3, and G16 were isolated from the rhizosphere soil of wild *Medicago sativa* in the Pisha sandstone region.

### 3.2 16S rRNA gene sequence analysis of selected strains

Genomic DNA was first extracted from the selected bacterial strains, followed by PCR amplification of the 16S rRNA gene using universal bacterial primers F27 and R1492. The amplified products from all nine strains were sent to a commercial sequencing facility for bidirectional sequencing.

The obtained sequences were analyzed using the NCBI BLAST tool to identify their closest phylogenetic relatives. The results indicated that strain G16 belongs to the genus Acinetobacter; strains F6, F11, G1, and G3 to the genus Klebsiella; strain P9 to the genus Serratia; strain F7 to the genus Pseudomonas; and strain P12 to the genus Herbaspirillum.

**Figure 2:**
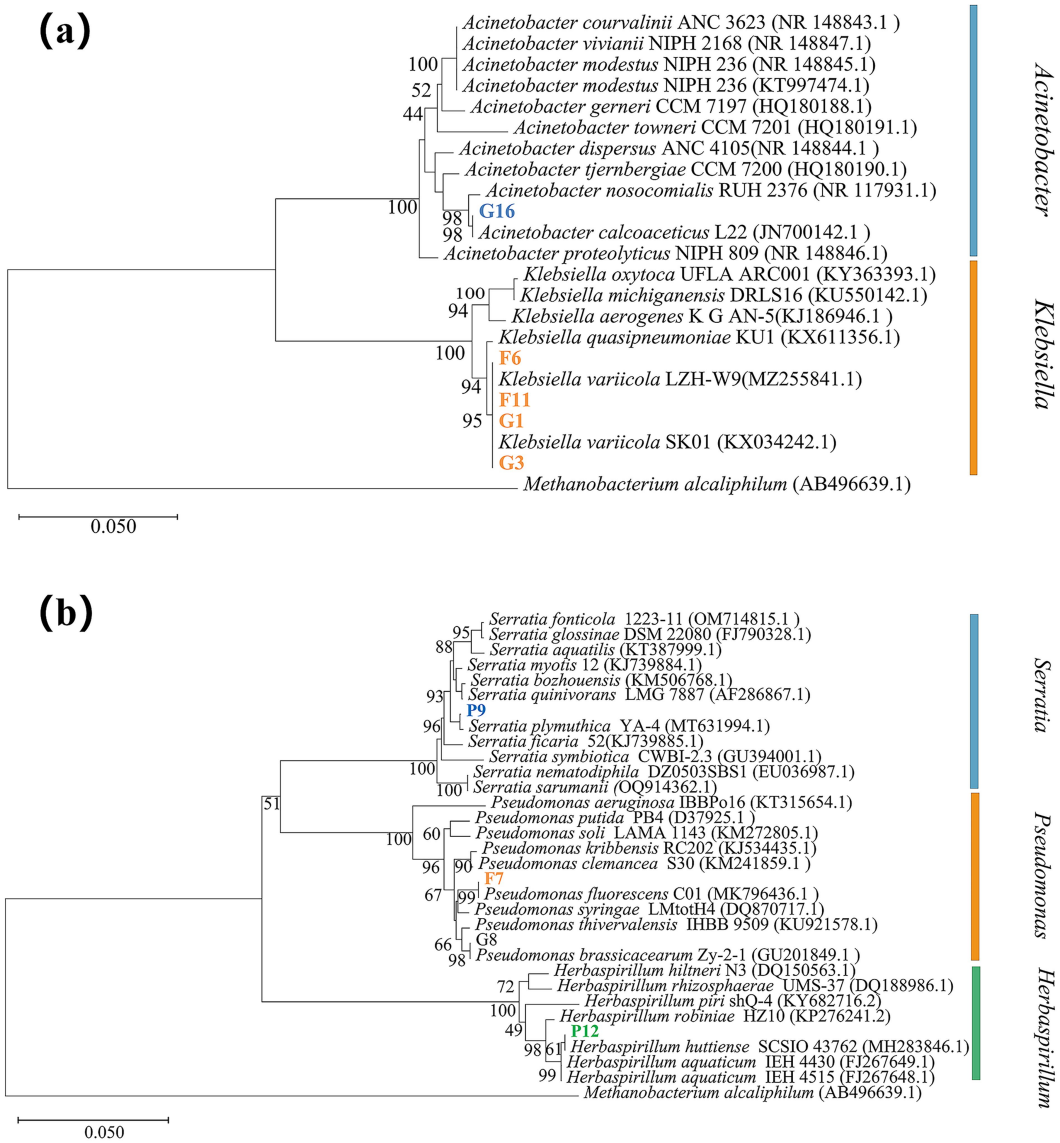
Phylogenetic tree based on 16S rDNA sequences of the selected bacterial strains. Note: Strains in bold represent the selected test strains, while the others are reference type strains. The alphanumeric codes in parentheses indicate the GenBank accession numbers of the corresponding 16S rDNA or gene sequences. Branch lengths represent sequence homology, and the numbers at the nodes indicate bootstrap support values. **(a)** Phylogenetic tree of strains G16, F6, F11, G1, and G3. **(b)** Phylogenetic tree of strains P9, F7, and P12.

A phylogenetic tree was constructed using MEGA X software, as shown in Figure 2.

Using *Methanobacterium alcaliphilum* (AB496639.1) as the outgroup reference strain, Figure 2a shows that strain G16 shares 99.86% sequence identity with *Acinetobacter calcoaceticus*, specifically aligning with *Acinetobacter calcoaceticus* L22. Strains F6, F11, G1, and G3 exhibit similar sequence homology to *Klebsiella* var*iicola*.

As shown in Figure 2b, strain P9 shares 99.79% sequence identity with *Serratia plymuthica*; strain F7 shows 100% identity with *Pseudomonas fluorescens*, aligning specifically with *Pseudomonas fluorescens* C01; and strain P12 shares 99.93% sequence identity with *Herbaspirillum huttiense*, corresponding to *Herbaspirillum huttiense* SCSIO 43762.

### 3.3 Screening and functional characterization of PGPB isolated from Pisha sandstone

A total of eight PGPB strains isolated from Pisha sandstone were evaluated for their key plant growth-promoting traits, including phosphorus solubilization (organic and inorganic), nitrogenase activity, IAA production, and siderophore secretion (Table 1). Significant differences (*p* < 0.05) were observed among strains for each trait.

**Table 1:** Functional traits of PGPB strains isolated from Pisha sandstone, including phosphorus solubilization, nitrogenase activity, IAA production, and siderophore production.

Strain number	Solubilized organic phosphorus content (mg/L)	Solubilized inorganic phosphorus content (mg/L)	Nitrogenase activity (U/L)	IAA content (mg/L)	Siderophore unit
P9	187.23 ± 1.54c	117.50 ± 3.73e	211.20 ± 1.04b	4.50 ± 0.06ef	81.8%
P12	112.79 ± 2.65 g	9.80 ± 0.33i	116.65 ± 1.95 g	5.89 ± 0.51e	47.1%
F6	283.19 ± 0.90a	146.15 ± 2.30b	214.39 ± 2.45b	86.44 ± 0.26a	85.6%
F7	153.59 ± 0.87e	62.58 ± 0.83f	192.59 ± 2.91c	8.72 ± 0.06d	31.2%
F11	198.29 ± 0.62b	126.19 ± 1.81d	171.17 ± 2.85d	67.99 ± 0.24b	68.7%
G1	184.31 ± 2.02c	138.86 ± 3.30c	224.46 ± 0.80a	88.13 ± 0.12a	82.1%
G3	183.97 ± 2.67c	182.44 ± 1.47a	127.84 ± 2.71f	59.58 ± 0.43c	−0.9%
G16	145.98 ± 0.90f	35.63 ± 2.24 g	141.91 ± 3.08e	1.83 ± 0.01f	79.5%

Data are means ± standard error (*n* = 3). Different lowercase letters indicate statistically significant differences among treatments for the same plant species according to one-way ANOVA followed by Duncan’s multiple range test (*p* < 0.05).

Strain F6 exhibited the highest level of organic phosphorus solubilization (283.19 ± 0.90 mg/L), as well as high inorganic phosphorus solubilization (146.15 ± 2.30 mg/L) and nitrogenase activity (214.39 ± 2.45 U/L). It also produced a high concentration of IAA (86.44 ± 0.26 mg/L) and siderophores (85.6%). Similarly, G1 showed strong performance across all traits, with the highest nitrogenase activity (224.46 ± 0.80 U/L) and IAA production (88.13 ± 0.12 mg/L), and notable phosphorus solubilizing and siderophore-producing capacities.

Strains F11, P9, and G3 also demonstrated promising functional characteristics. F11 showed balanced performance with strong organic and inorganic phosphorus solubilization and high IAA production (67.99 ± 0.24 mg/L). P9 exhibited high organic phosphorus solubilization (187.23 ± 1.54 mg/L) and nitrogenase activity (211.20 ± 1.04 U/L), while G3 had the highest inorganic phosphorus solubilization capacity (182.44 ± 1.47 mg/L), despite showing negative siderophore activity.

Considering all the above plant growth-promoting traits, five strains-P9, F6, F11, G1, and G3-were selected for further investigation. These were designated as follows: F6 as bacterial suspension 1, F11 as bacterial suspension 2, G1 as bacterial suspension 3, G3 as bacterial suspension 4, and P9 as bacterial suspension 5, for use in subsequent experiments.

### 3.4 Effects of bacterial suspensions on soil nutrients and plant growth in Pisha sandstone soil

**Figure 3:**
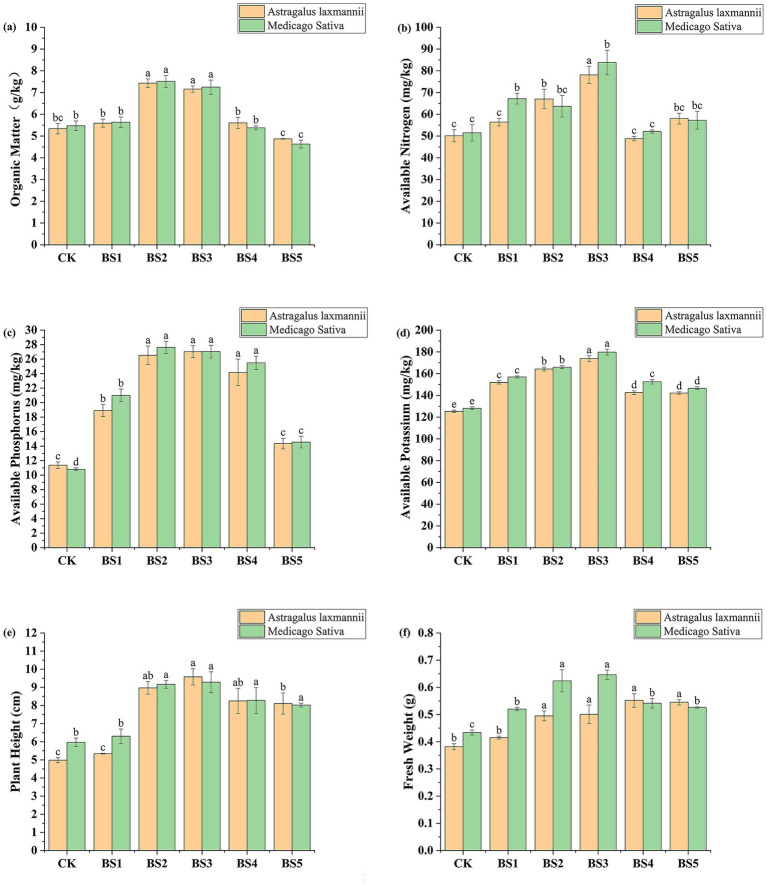
Effects of bacterial suspensions on soil nutrient availability and plant growth parameters in Pisha sandstone soils. Panels show changes in **(a)** soil organic matter, **(b)** available nitrogen, **(c)** available phosphorus, **(d)** available potassium, **(e)** plant height, and **(f)** fresh biomass of *Astragalus laxmannii* and *Medicago sativa* following inoculation with different plant growth-promoting bacteria (PGPB). Note: CK denotes the uninoculated control. BS1–BS5 represent bacterial suspensions prepared from strains F6, F11, G1, G3, and P9, respectively. Data are presented as means ± standard error (*n* = 3). Different lowercase letters indicate statistically significant differences among treatments for the same plant species according to one-way ANOVA followed by Duncan’s multiple range test (*p* < 0.05).

To evaluate the effects of selected PGPB strains on plant growth under nutrient-poor conditions, a pot experiment was conducted with *Astragalus laxmannii* and *Medicago sativa*. Compared with the uninoculated control (CK), the bacterial suspensions (BS1-BS5) significantly improved soil fertility and plant growth parameters, as shown in Figure 3.

For *Astragalus laxmannii*, BS3 (strain G1) resulted in the highest available nitrogen content of 78.11 mg/kg, which represents an increase of 55.8% compared to CK (50.12 mg/kg). Available phosphorus and potassium under BS3 reached 27.02 and 173.74 mg/kg, representing increases of 137.6 and 38.6%, respectively, compared to CK (11.37 and 125.30 mg/kg). The highest soil organic matter content was observed under BS2 (strain F11) with 7.43 g/kg, increased by 39.2% compared to CK (5.34 g/kg).

For *Medicago sativa*, BS3 similarly showed the greatest improvement. Available nitrogen, phosphorus, and potassium contents were 83.77, 27.03, and 179.72 mg/kg, which corresponded to increases of 62.7, 150.2, and 40.2%relative to CK (51.48, 10.80, and 128.12 mg/kg), respectively. Soil organic matter under BS2 increased by 37.3% to 7.51 g/kg compared to CK (5.47 g/kg).

Regarding plant growth, BS3 significantly enhanced both plant height and fresh weight. For *Astragalus laxmannii*, plant height increased from 4.98 cm in CK to 9.58 cm, an increase of 92.3%, while fresh weight increased by 31.0% from 0.382 g to 0.5007 g. For *Medicago sativa*, plant height increased by 55.7% from 5.96 cm to 9.28 cm, and fresh weight increased by 49.0% from 0.4337 g to 0.6463 g. BS2 also notably increased fresh weight, with *Astragalus laxmannii* increasing by 29.6% to 0.495 g and *Medicago sativa* by 43.9% to 0.624 g compared to CK. BS5 and CK generally showed the lowest values across all parameters.

These results demonstrate that specific PGPB strains, particularly G1 (BS3) and F11 (BS2), can effectively enhance nutrient availability and promote legume growth in nutrient-deficient Pisha sandstone soils, indicating strong potential for ecological restoration applications.

### 3.5 Effects of microbial inoculants on soil nutrients, enzyme activities, and plant growth in Pisha sandstone soil

Based on a comprehensive evaluation of earlier plant growth-promoting traits, strains F11 and G1 were selected for the development of microbial inoculants. Three formulations were prepared: microbial inoculant 1 (MI1) using strain F11, microbial inoculant 2 (MI2) using strain G1, and microbial inoculant 3 (MI3) combining both in equal volumes. The control group (CK1) received only the inoculant carrier at the same 5% application rate but without any microbial strain.

#### 3.5.1 Improvements in soil nutrient content and organic matter

**Figure 4:**
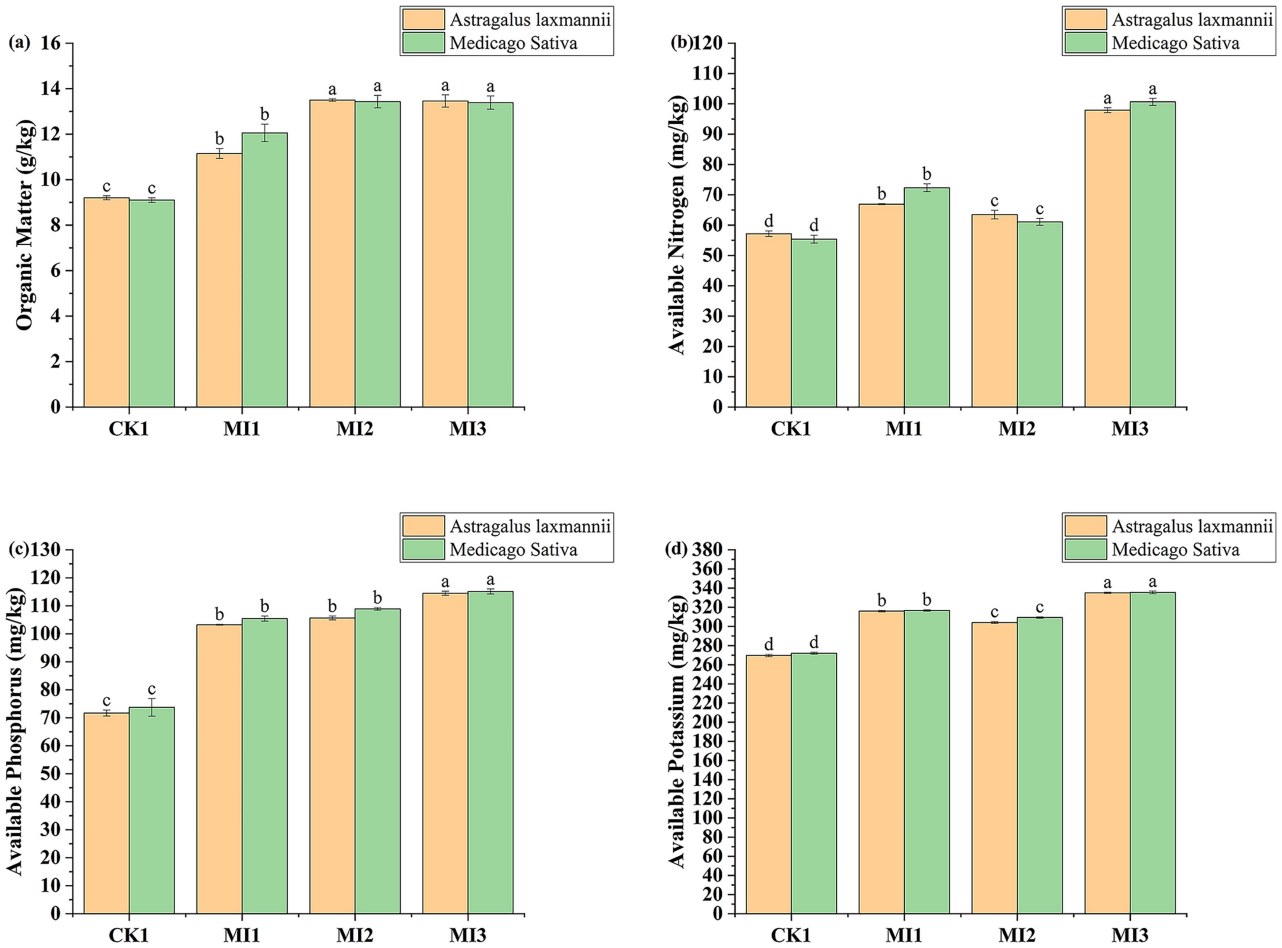
Effects of microbial inoculants on soil nutrient availability in Pisha sandstone soils. Panels show changes in **(a)** available nitrogen, **(b)** available phosphorus, **(c)** available potassium, and **(d)** soil organic matter under cultivation of *Astragalus laxmannii* and *Medicago sativa*. Data are means ± standard error (*n* = 3). Different lowercase letters indicate statistically significant differences among treatments for the same plant species according to one-way ANOVA followed by Duncan’s multiple range test (*p* < 0.05).

The application of microbial inoculants significantly enhanced the concentrations of available nitrogen, phosphorus, potassium, and organic matter in the Pisha sandstone soil (Figure 4). For *Astragalus laxmannii*, the MI3 treatment increased available nitrogen from 57.15 mg/kg (CK1) to 97.89 mg/kg, representing a 71.3% improvement. Available phosphorus increased by 59.7% (from 71.67 to 114.46 mg/kg), potassium by 24.3% (from 269.59 to 335.03 mg/kg), and organic matter content by 46.3% (from 9.20 to 13.46 g/kg).

In *Medicago sativa*-planted soil, MI3 also led to substantial increases: available nitrogen rose from 55.38 mg/kg to 100.65 mg/kg (81.8% increase), phosphorus from 73.71 to 115.12 mg/kg (56.2%), potassium from 272.02 to 335.51 mg/kg (23.3%), and organic matter from 9.10 to 13.39 g/kg (47.1%).

Further comparisons among treatments revealed that MI2 (strain G1) and MI1 (strain F11) also improved soil fertility, but to a lesser extent than MI3. Specifically, MI2 raised available nitrogen to 63.47 mg/kg in *Astragalus laxmannii* soil (an increase of 11.1%) and to 61.07 mg/kg in *Medicago sativa* soil (10.3%). Available phosphorus increased by 47.4 and 47.7% in *Astragalus laxmannii* and *Medicago sativa* soils, respectively, while potassium content increased by 12.8 and 13.7%.

Organic matter content under MI2 also showed notable enhancement, reaching 13.50 g/kg in *Astragalus laxmannii*-planted soil and 13.43 g/kg in *Medicago sativa*-planted soil, improvements of 46.7 and 47.5% compared to CK1. In contrast, MI1 yielded more modest improvements: nitrogen increased by 17.0% in *Astragalus laxmannii* soil and 30.6% in *Medicago sativa* soil; phosphorus by 44.0 and 43.0%; potassium by 17.2 and 16.4%; and organic matter content by 21.2 and 32.4%, respectively.

These results demonstrate that microbial inoculants, especially MI3, not only promote nutrient cycling in Pisha sandstone soil but also improve organic matter accumulation, with particularly strong effects when both F11 and G1 strains are co-applied.

#### 3.5.2 Enhancements in plant height and biomass

**Figure 5:**
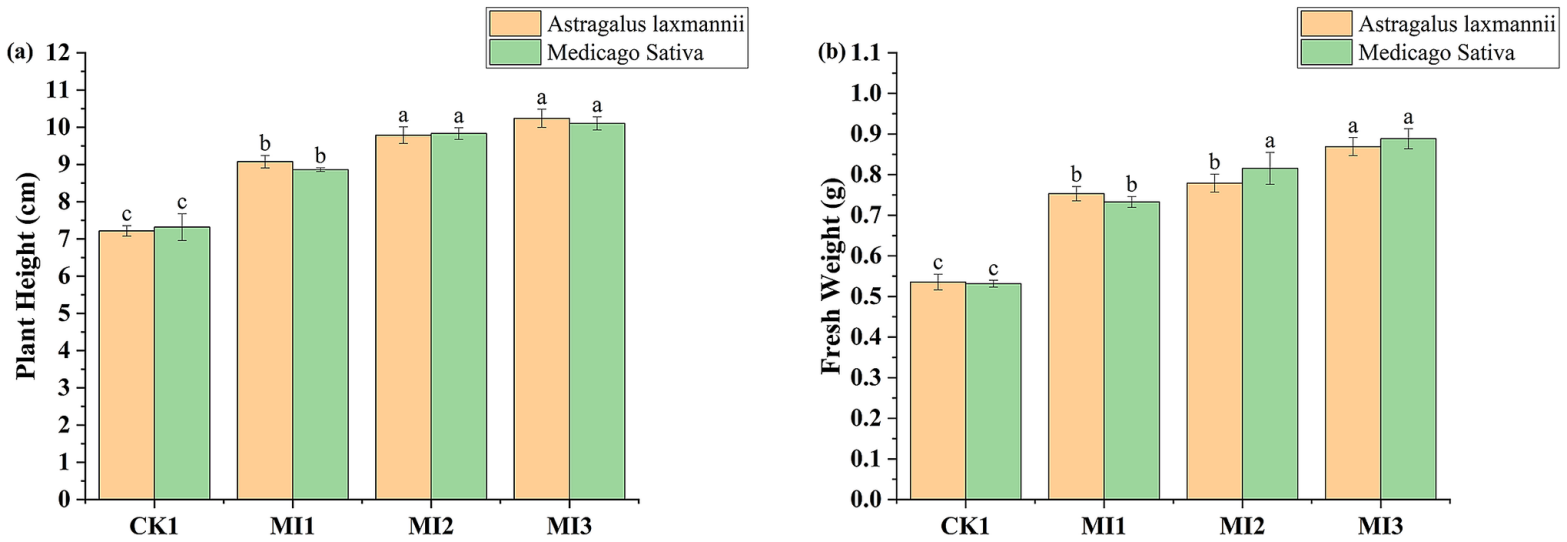
Effects of microbial inoculants on plant growth parameters in Pisha sandstone soils. Panels show changes in **(a)** plant height and **(b)** fresh biomass of *Astragalus laxmannii* and *Medicago sativa* following inoculation. Data are means ± standard error (*n* = 3). Different lowercase letters indicate statistically significant differences among treatments for the same plant species according to one-way ANOVA followed by Duncan’s multiple range test (*p* < 0.05).

Microbial inoculant treatments substantially improved plant height and fresh weight compared to the control (Figure 5). For *Astragalus laxmannii*, MI3 increased plant height from 7.21 cm to 10.24 cm (41.9% increase) and fresh weight from 0.535 g to 0.869 g (62.3%). For *Medicago sativa*, plant height increased from 7.32 cm (CK1) to 10.10 cm (MI3), a 38.0% increase, while fresh weight improved from 0.531 g to 0.888 g (67.2%).

#### 3.5.3 Stimulation of soil enzyme activities

**Figure 6:**
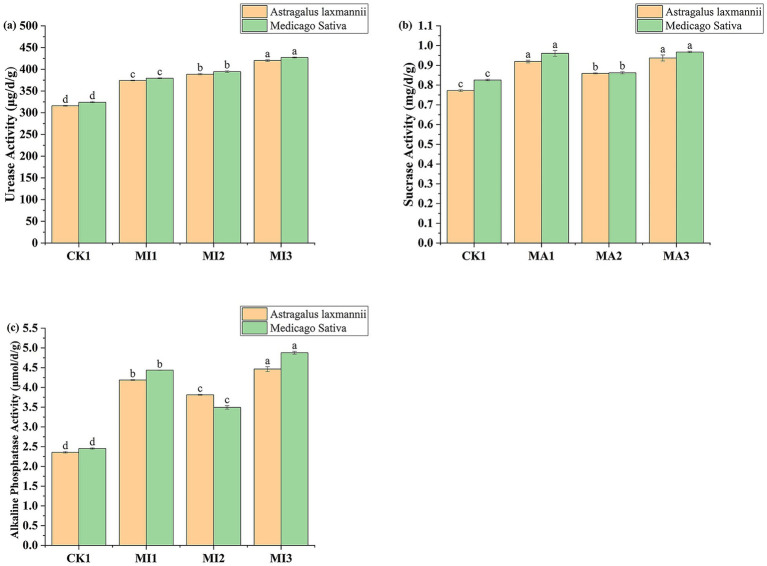
Effects of microbial inoculants on soil enzyme activities in Pisha sandstone soils. Panels show activities of **(a)** urease, **(b)** sucrase, and **(c)** alkaline phosphatase in soils cultivated with *Astragalus laxmannii* and *Medicago sativa*. Different lowercase letters indicate statistically significant differences among treatments for the same plant species according to one-way ANOVA followed by Duncan’s multiple range test (*p* < 0.05).

Soil enzyme activities were also significantly affected by microbial inoculant treatments (Figure 6). In soils planted with *Astragalus laxmannii*, urease activity increased from 315.97 μg/d/g in CK1 to 374.22, 388.61, and 419.95 μg/d/g in MI1, MI2, and MI3 treatments, representing relative increases of 18.4, 23.0, and 32.9%, respectively. Similarly, in soils planted with *Medicago sativa*, urease activity rose from 324.15 μg/d/g in CK1 to 379.44, 394.54, and 427.05 μg/d/g under MI1, MI2, and MI3, corresponding to increases of 17.1, 21.7, and 31.8%. Overall, urease activity was slightly higher in soils planted with *Medicago sativa* than with *Astragalus laxmannii* under CK1, MI1, and MI3, while the reverse was observed under MI2.

Sucrase activity followed a similar pattern. In *Astragalus laxmannii* soils, the activity increased from 0.772 mg/d/g in CK1 to 0.919, 0.859, and 0.937 mg/d/g in MI1, MI2, and MI3, reflecting enhancements of 19.0, 11.3, and 21.4%, respectively. In *Medicago sativa* soils, sucrase activity rose from 0.825 mg/d/g in CK1 to 0.961, 0.862, and 0.967 mg/d/g under MI1, MI2, and MI3, corresponding to increases of 16.4, 4.5, and 17.2%. Notably, sucrase activity was generally higher in *Astragalus laxmannii*-planted soils under CK1, MI1, and MI3 treatments, while activity was similar across both plants under MI2.

Alkaline phosphatase activity was most strongly affected by MI3. In *Astragalus laxmannii*-planted soils, activity increased from 2.36 μmol/d/g in CK1 to 4.19, 3.81, and 4.47 μmol/d/g under MI1, MI2, and MI3, resulting in increases of 77.8, 61.7, and 89.7%, respectively. For *Medicago sativa*, activity increased from 2.45 μmol/d/g in CK1 to 4.44, 3.49, and 4.88 μmol/d/g in MI1, MI2, and MI3, reflecting enhancements of 81.0, 42.4, and 99.0%. MI3 significantly outperformed other treatments for both crops, and phosphatase activity was consistently higher in *Astragalus laxmannii*-planted soils under MI3 treatment.

These results collectively indicate that microbial inoculants, particularly the combined treatment MI3, substantially enhanced soil enzyme activities, contributing to improved nutrient cycling in Pisha sandstone soils.

## 4 Discussion

In this study, PGPB strains were isolated from degraded soils in the Pisha sandstone region. Through systematic isolation, functional screening, and validation via pot experiments, several promising strains capable of enhancing soil fertility and promoting plant growth were identified. Based on the experimental findings, a rational microbial inoculation strategy was formulated. Ultimately, effective microbial inoculants were selected that significantly improved both soil quality and plant performance, offering theoretical guidance and technical support for soil restoration and sustainable agricultural development in ecologically fragile areas.

### 4.1 Strain selection and growth-promotion mechanisms

In this study, the isolated strains exhibited multiple plant growth-promoting traits that could play key roles in soil improvement and vegetation restoration in the Pisha sandstone area. Several strains demonstrated strong phosphate-solubilizing abilities, with F6 and G3 showing the highest performance on inorganic phosphate, while F6 also achieved the greatest solubilization of organic phosphate. These results suggest that such strains may enhance nutrient availability for plants by mobilizing phosphorus from otherwise insoluble sources, which is consistent with previous findings that phosphate-solubilizing bacteria contribute to soil fertility improvement (Timofeeva et al., 2022; Khan et al., 2025). In terms of nitrogen metabolism, strain G1 exhibited the highest nitrogenase activity (224.46 U/L), significantly higher than reported in other studies (Li H. et al., 2020), indicating its strong potential to supplement nitrogen in nutrient-deficient soils. Furthermore, most isolates produced high levels of indole-3-acetic acid (IAA), with G1 and F6 reaching nearly 90 mg/L, implying their ability to stimulate root development and increase nutrient and water uptake efficiency (John et al., 2020; Jalmi and Sinha, 2022). In addition, siderophore production was particularly pronounced in F6 and G1, reaching 85.6 and 82.1%, respectively, which may help plants adapt to iron-limited conditions (Santoyo et al., 2021; Gao et al., 2022; Brick et al., 2025). Taken together, these functional traits highlight the synergistic mechanisms through which the selected strains can promote plant growth and enhance soil quality.

Based on 16S rRNA sequencing and phylogenetic analysis, the selected strains belong to typical PGPB genera, including *Klebsiella* var*iicola*, *Acinetobacter calcoaceticus*, *Pseudomonas fluorescens*, *Serratia plymuthica*, and *Herbaspirillum huttiense*, all known for phosphate solubilization, nitrogen fixation, and IAA production (Andreozzi et al., 2019; Kwaslema et al., 2022; Prajapati et al., 2022). The consistency between the observed plant growth-promoting traits and the well-documented functional capacities of these genera further confirms the robustness of the isolated strains as potential bioinoculants.

These findings underscore the importance of integrating both functional and phylogenetic characterization when evaluating PGPB for ecological restoration. The combined application of these strains may therefore represent a promising strategy for enhancing soil fertility, promoting sustainable vegetation establishment, and ultimately accelerating the rehabilitation of degraded Pisha sandstone ecosystems.

### 4.2 Effects of bacterial suspensions on soil nutrients and plant growth

The application of bacterial suspensions significantly improved soil fertility in the nutrient-poor Pisha sandstone soils, as reflected by increases in soil organic matter (SOM) and the availability of nitrogen, phosphorus, and potassium. SOM is a key reservoir of carbon, essential for aggregate stability, soil structure improvement, and nutrient supply (Reeves, 1997; Johannes et al., 2023). In this study, inoculation with F11 (BS2) increased SOM by over 39% compared to the control, which is consistent with reports that PGPB inoculation can accelerate organic matter accumulation by stimulating root exudation and enhancing microbial biomass (Meier et al., 2017; Maurer et al., 2021). Enhanced SOM contributes not only to nutrient retention but also to improved water-holding capacity, which is particularly critical in the fragile and erosion-prone soils of the Pisha sandstone region.

Nitrogen, phosphorus, and potassium are essential macronutrients for plant growth, each playing a distinct physiological role: nitrogen is central to protein synthesis and photosynthesis (Bassi et al., 2018); phosphorus is critical for energy transfer, root growth, and genetic material (Delfim et al., 2024); potassium regulates osmotic pressure, turgor, membrane potential, protein synthesis, and assimilate transport, comprising 2–10% of plant dry weight (Leigh and Jones, 1984; Zörb et al., 2014; Almeida et al., 2015). In this study, G1 (BS3) achieved the most pronounced effects on nutrient availability, with soil available nitrogen, phosphorus, and potassium increasing by up to 62.7, 150.2, and 40.2%, respectively. These findings align with previous studies showing that PGPB can improve nutrient cycling through multiple mechanisms, including nitrogen fixation, phosphate solubilization, and potassium mobilization (Bhattacharyya and Furtak, 2023; Cao et al., 2024).

The improved soil fertility directly translated into enhanced plant performance. Both *Astragalus laxmannii* and *Medicago sativa* exhibited substantial increases in plant height and biomass under PGPB treatments, with BS3 and BS2 being the most effective. These results are in agreement with earlier findings that PGPB inoculation enhances legume growth by promoting nutrient acquisition and hormonal stimulation (Swarnalakshmi et al., 2020). Notably, legumes such as *Astragalus laxmannii* and *Medicago sativa* also engage in symbiotic nitrogen fixation, and PGPB inoculation may further complement this process by improving root growth and facilitating nodulation (Bauer et al., 2012; Cangioli et al., 2025).

Taken together, the observed improvements in SOM, nutrient availability, and plant growth demonstrate that selected PGPB strains, particularly G1 and F11, are promising candidates for bioinoculant development. Their application could play a vital role in restoring the fertility and productivity of degraded Pisha sandstone soils, while also contributing to sustainable vegetation establishment in ecologically fragile landscapes.

### 4.3 Microbial inoculants and their effects on Pisha sandstone and plant growth

Biochar possesses favorable physical structure and chemical properties, making it an effective soil conditioner (Kambo and Dutta, 2015). It not only reduces nutrient leaching (Baiamonte et al., 2015) but also improves soil structure and provides a suitable habitat for microorganisms, thereby promoting the growth of beneficial microbes (Lehmann, 2007). Its porous architecture, especially macropores, facilitates the colonization and proliferation of microorganisms such as *Trichoderma* spp., while supplying carbon sources and minerals that enhance microbial stability and functionality (Paveen et al., 2025). Meanwhile, sheep manure, as a source of organic matter, is rich in nitrogen, phosphorus, and potassium nutrients, which provide essential nourishment for microbial growth. Additionally, the synergistic interaction between organic substrates and biochar may regulate soil pH, improve soil aggregate structure, and increase cation exchange capacity (Ibrahim et al., 2020), thereby enhancing interactions between microorganisms and the plant rhizosphere. Based on this, a microbial carrier was prepared by mixing sheep manure and straw biochar at a mass ratio of 2:1, to which microbial strains were added to formulate microbial inoculants for a second round of pot experiments.

Both the application of bacterial suspensions and microbial inoculants significantly improved soil quality and plant performance. However, microbial inoculants, particularly MI3 (containing strains F11 and G1), demonstrated superior efficacy. For instance, MI3 increased soil organic matter content to 13.50 g/kg, compared to 7.43 g/kg under BS2 (strain F11 suspension), representing respective increases of 46.3 and 39.14% over the uninoculated control. Similarly, MI3 enhanced available nitrogen levels by 71.3–81.8%, whereas the best-performing suspension (BS3) achieved 55.85–62.72% increases. Available phosphorus and potassium were also more effectively elevated by MI3, with phosphorus increasing by 59.7–56.2% and potassium by 24.3--23.3%, compared to lower gains under bacterial suspension treatments.

Notably, microbial inoculants significantly enhanced soil enzymatic activity. Urease activity under MI3 reached 419.95 μg/d/g and 427.05 μg/d/g in soils planted with *Astragalus laxmannii* and *Medicago sativa*, respectively-substantially higher than in soils treated with bacterial suspensions. Sucrase and alkaline phosphatase activities followed similar trends, indicating that microbial inoculants not only improve nutrient levels but also modulate soil biochemical processes more effectively than bacterial suspensions (Khan et al., 2023; Hao et al., 2025). These outcomes likely result from a combination of direct metabolic activity by inoculated strains and their synergistic effects on indigenous microbial communities.

In terms of plant growth, MI3 significantly outperformed bacterial suspensions. It increased the height of *Astragalus laxmannii* and *Medicago sativa* by 42.02 and 38.36%, respectively, and their fresh biomass by 61.11 and 67.92%, respectively. By comparison, bacterial suspensions led to more modest increases in these parameters.

In summary, these findings highlight the superior performance of microbial inoculants over bacterial suspensions in enhancing soil fertility, stimulating enzymatic activity, and promoting plant growth in degraded Pisha sandstone soils. The enhanced efficacy of microbial inoculants may be attributed to their improved environmental stability, sustained microbial viability, and stronger interaction with the soil matrix, making them a promising strategy for ecological restoration and sustainable agriculture in fragile landscapes.

## 5 Conclusion

In this study, five functional PGPB strains—F6, F11, G1, G3, and P9—were successfully isolated from Pisha sandstone soils. These strains exhibited distinct plant growth-promoting traits, including phosphorus solubilization (both inorganic and organic), nitrogen fixation, and the production of IAA and siderophores. Further studies on strain combinations and suspension formulations revealed that under the MI3 treatment condition (a combination of G1 and F11), plant height and biomass increased by up to 47.2 and 52.6%, respectively, compared to the control. Based on these results, a composite microbial inoculant was developed using sheep manure-straw biochar as a carrier, and its application significantly improved the physicochemical and biological properties of Pisha sandstone soils. Soil nutrient levels and enzyme activities were notably enhanced, while the plant growth of *Medicago sativa* and *Astragalus laxmannii* was effectively promoted. Although these findings confirm the potential of PGPB-based inoculants for Pisha sandstone improvement, they were obtained from controlled pot experiments. Field validation in the Erlahugou demonstration area is already in preparation and will be essential to confirm their long-term effectiveness and ecological applicability.

## Data Availability

All sequence data have been deposited in GenBank. The accession numbers are as follows: Strain G1: PV689977 Strain F11: PV690022 Strain G16: PX232812 Strain P12: PX232708 Strain P9: PX232707 Strain G3: PX232665 Strain F7: PX232663 Strain F6: PX229914. The datasets generated, used, and/or analyzed during the current study are available from the corresponding author upon reasonable request.
